# Genome analysis of a wild rumen bacterium *Enterobacter aerogenes* LU2 - a novel bio-based succinic acid producer

**DOI:** 10.1038/s41598-020-58929-0

**Published:** 2020-02-06

**Authors:** Hubert Szczerba, Elwira Komoń-Janczara, Mariusz Krawczyk, Karolina Dudziak, Anna Nowak, Adam Kuzdraliński, Adam Waśko, Zdzisław Targoński

**Affiliations:** 1 Department of Biotechnology, Microbiology and Human Nutrition, University of Life Sciences in Lublin, 8 Skromna Street, 20-704 Lublin, Poland; 2 Genomed SA, 12 Ponczowa Street, 02-971 Warsaw, Poland; 3 Chair and Department of Biochemistry and Molecular Biology, Medical University of Lublin, 1 Chodźki Street, 20-093 Lublin, Poland

**Keywords:** Applied microbiology, Industrial microbiology

## Abstract

*Enterobacter aerogenes* LU2 was isolated from cow rumen and recognized as a potential succinic acid producer in our previous study. Here, we present the first complete genome sequence of this new, wild strain and report its basic genetic features from a biotechnological perspective. The MinION single-molecule nanopore sequencer supported by the Illumina MiSeq platform yielded a circular 5,062,651 bp chromosome with a GC content of 55% that lacked plasmids. A total of 4,986 genes, including 4,741 protein-coding genes, 22 rRNA-, 86 tRNA-, and 10 ncRNA-encoding genes and 127 pseudogenes, were predicted. The genome features of the studied strain and other *Enterobacteriaceae* strains were compared. Functional studies on the genome content, metabolic pathways, growth, and carbon transport and utilization were performed. The genomic analysis indicates that succinic acid can be produced by the LU2 strain through the reductive branch of the tricarboxylic acid cycle (TCA) and the glyoxylate pathway. Antibiotic resistance genes were determined, and the potential for bacteriocin production was verified. Furthermore, one intact prophage region of length ~31,9 kb, 47 genomic islands (GIs) and many insertion sequences (ISs) as well as tandem repeats (TRs) were identified. No clustered regularly interspaced short palindromic repeats (CRISPRs) were found. Finally, comparative genome analysis with well-known succinic acid producers was conducted. The genome sequence illustrates that the LU2 strain has several desirable traits, which confirm its potential to be a highly efficient platform for the production of bulk chemicals.

## Introduction

*Enterobacter aerogenes* LU2 is a gram-negative, wild bacterium that was isolated from cow rumen as a part of environmental screening for succinic acid-producing bacteria identification. Succinic acid (SA) plays an important role as a metabolic intermediate in the rumen by increasing propionate production, a crucial energy source for the ruminant^1,2^. Anaerobic conditions and the presence of carbon dioxide, methane and trace amounts of hydrogen create an excellent environment for the biosynthesis of succinate by some bacteria existing in the rumen^3^.

In reports of the U.S. Department of Energy (DOE) from 2004 and 2010, SA was recognized as one of the top 10 most promising C4-building chemical platforms for the production of high-value commodity and specialty chemicals with great industrial potential^2,4^. Succinate is widely applied as an additive in food, pharmaceuticals, detergents, solvents, and surfactants as well as in biodegradable polymer production^5,6^.

Until recently, industrial production of succinate was based on chemical synthesis. However, petroleum-based production of SA from *n*-butane through maleic anhydrate requires the use of high pressure, high temperature and costly catalysts^7^. Therefore, because of the current global trend regarding sustainable development, including the support of green technologies, rational waste biomass management and pollution-reducing standards, the bio-based production of succinate by highly efficient microbial producers from renewable feedstocks has become a point of global interest^8^.

Among the most frequently described bacterial species that have been recognized as natural succinic acid producers are *Basfia succiniciproducens, Mannheimia succiniciproducens*, *Actinobacillus succinogenes* and *Anaerobiospirillum succiniciproducens*^9–12^. These microorganisms are well characterized both physiologically and biochemically. Moreover, complete genome sequences of these bacteria have already been obtained, enabling further study in metabolic engineering. Meanwhile, *Enterobacter* species are still an undiscovered source of new strains that are predisposed to efficient production of succinate.

In our previous study, the identification and physiological characterization of *E*. *aerogenes* LU2 were performed. The novelty and attractiveness of this new strain is that it is the first wild strain from the species *Enterobacter aerogenes* that is capable of efficiently producing SA by utilizing whey permeate with high lactose content derived from the whey industry. However, to better understand the unique properties of this strain, in this study, a complete genome was obtained, and genetic characterization was performed.

Several draft genomes of potential bacterial producers of organic acids that have a broad range of industry applications have been sequenced, mainly using short-read technologies (Illumina, 454 Life Sciences)^13–16^. Consequently, many genomes are incomplete multi-contig builds, probably due to the use of only short reads, making complete genome assembly difficult to perform and hindering comparative genomic studies^17^. In contrast, the long-read platform (Oxford Nanopore Technologies, ONT) is characterized by a higher cost per base and has much higher per-base error rates in comparison with those of the Illumina sequencing platform^18^. Combining these two technologies is, accordingly, a great approach that provides accurate contigs and the information necessary to scaffold them together from the short and long reads, respectively.

Here, we report the first complete genome sequence and detailed genomic analysis of *Enterobacter aerogenes* LU2 established by highly efficient and cost-effective hybrid sequencing, including long-read single-molecule nanopore sequencing by ONT MinION paired with the short-read Illumina MiSeq platform.

## Results and Discussion

### Genome features of *Enterobacter aerogenes* LU2

**Figure 1:**
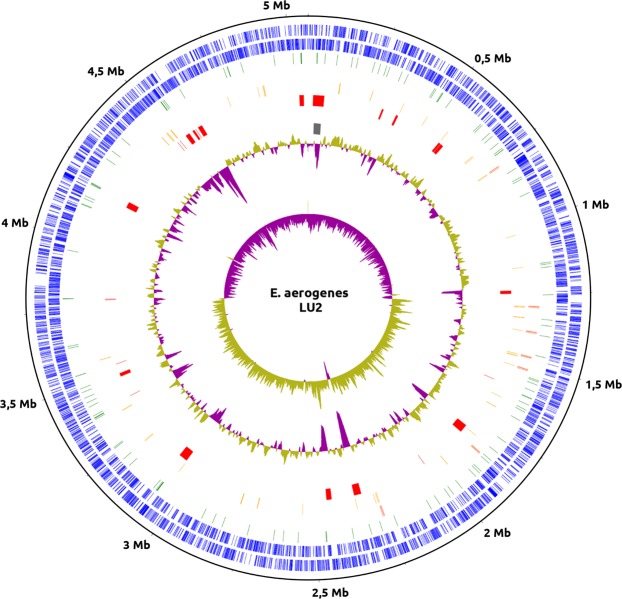
Circular map of *Enterobacter aerogenes* LU2 genome. From outer circle to inner circle, representation is as follows: 1. Position in megabases (black); 2. Forward strand CDSs (blue); 3. Reverse strand CDSs (blue); 4. Pseudogenes (green); 5. rRNAs (dark orange); 6. tRNAs (orange); 7. Horizontal gene transfer (HGT) regions (red); 8. Phage sequences (grey); 9. GC content (mustard and purple color correspond to higher and lower than average GC content, respectively); 10. GC skew (mustard and purple color correspond to higher and lower than average GC skew, respectively). The whole-genome sequence visualization was performed using CGView software, http://wishart.biology.ualberta.ca/cgview/.

A single circular chromosome of 5,062,651 bp in size with no plasmids was obtained (Fig. 1). The overall GC content of the chromosome amounted to 55%. A GC skew transition was clearly observed, and *oriC* (origin of replication) and *terC* (terminus of replication) were identified at the positions of 1,280,000 and 3,793,000, respectively. A total of 4986 genes have been annotated in the genome, of which 4741 are protein-coding genes. Furthermore, 22 rRNA-, 86 tRNA-, and 10 ncRNA-encoding genes and 127 pseudogenes were predicted. The number of rRNAs and tRNAs in the LU2 strain is comparable with that of other strains listed in Table 1 and may indicate positive selection. A large number of rRNA genes is associated with a high activity of the translational apparatus, which in turn increases both the protein synthesis rate and the growth rate^19^. This phenomenon may be connected with strong competition among microorganisms located in the same ecological niche. The general features of the *E*. *aerogenes* LU2 genome compared to the genomes of the reference strain (*E*. *aerogenes* KCTC 2190) and ten other *Enterobacteriaceae* strains that have been retrieved from the NCBI GenBank database are summarized in Table 1. Compared with all twenty-two complete genome sequences of *E*. *aerogenes* species deposited in the NCBI GenBank database (access 25.11.2019), the LU2 strain genome is the smallest (Table 1, Additional file 1). It is also smaller than the average genome size of ten selected strains belonging to the *Enterobacteriaceae* family (Table 2, Additional file 1). However, the LU2 strain contains relatively many genes, resulting in the highest gene density (0,985 gene/kb) compared with that of other studied strains. Among the 4868 identified coding DNA sequences (CDSs), 4741 (97,4%) were classified as protein-coding genes, while only 127 (2,6%) were annotated as hypothetical proteins with unknown function. The total length of the protein-coding genes amounted to 4,813,884 bp, which is 95,09% of the whole genome sequence length, and the average gene length was 1015,4 bp.

**Table 1:** General features of *Enterobacter aerogenes* LU2 genome compared to reference genome (KCTC 2190) and ten^a^ complete *Enterobacteriaceae* genomes.

Genome features	*E. aerogenes* LU2	*E. aerogenes* KCTC 2190	*Enterobacteriaceae* average^b^
Genome size (bp)	5,062,651	5,280,350	5,129,521
Plasmids	0	0	1,5
Contigs	1	88	2,5
GC content (%)	55	54,8	53,23
Total genes	4,986	5,021	4998,9
Protein-coding genes	4,741	4,912	4750,8
Gene density (gene/kb)	0,985	0,930	0,975
5S rRNAs	8	25	23,5
16S rRNAs	7		
23S rRNAs	7		
tRNAs	86	84	83,4
ncRNAs	10	—	—
Pseudogenes	127	—	118,1
CRISPR arrays	0	—	—

“–”no data available.

^a^*Escherichia coli* AIA39, K12, NRG857c strains, *Citrobacter freundii* CFNIH1, FDAARGOS73, CAV1321 strains, *Raoultella ornithinolytica* B6, S12, A14 strains, *Enterobacter cloaceae* ATCC13047 strain.

^b^Data based on genome summary pages of NCBI GenBank database.

**Table 2:** Comparison of resistance related-genes presence in *Enterobacter aerogenes* LU2, KCTC 2190 and C10 strains.

Enzyme	Gene	Accession number of reference sequence	*Enterobacter aerogenes* strain		
			LU2	KCTC 2190	C10
Aminoglycoside N(3)-acetyltransferase III	aacC3	P0A255.1	−	−	+
ANT(3′′)-Ia family aminoglycoside nucleotidyltransferase	aadA25	WP_014325834.1	−	−	+
16S rRNA (adenine(1518)-N(6)/adenine(1519)-N(6))-dimethyltransferase	ksgA	WP_003829609.1	−	+	+
16S rRNA (guanine(1405)-N(7))-methyltransferase	rmtD	WP_019726361.1	−	−	−
AAC(6′)-Ib family aminoglycoside 6′-N-acetyltransferase	aacA4	WP_014839929.1 P19650.1	−	−	+
Chloramphenicol acetyltransferase 3	cat3	P00484.1	+	−	+
Beta-lactamase CTX-M-6	bla	O65976.1	−	−	+
Class A beta-lactamase - TEM family	bla	WP_010331504.1 WP_000027063.1	−	−	+
Class A beta-lactamase - TEM family	bla	WP_001398207.1	−	−	+
Ribonuclease Z/BN	rbn	WP_004890624.1	+	+	+
Class C beta-lactamase	N/A*	WP_008453751.1	+	+	+
Class D beta-lactamase (beta-lactamase OXA-2)	bla	P0A1V8.1	−	−	+
Class D beta-lactamase (oxacillinase-carbenicillinase OXA-9)	N/A	WP_004153119.1	−	−	+
Dihydropteroate synthase type-1	sulI	P0C002.1	+	+	+
Sulfonamide-resistant dihydropteroate synthase	sul2	WP_001043267.1	−	−	−
Trimethoprim-resistant dihydrofolate reductase	dfrA	WP_001611015.1	−	−	−
Undecaprenyl-diphosphatase	uppP	WP_012907642.1	+	+	+
Qnr family quinolone resistance pentapeptide repeat protein	qnr	WP_017111199.1	−	−	+
**Important feature**			Succinic acid producer	2,3-butanodiol producer	Human nosocomial pathogen
**Host**			Cow rumen	N/A	Hospitalized patients

*N/A- no data available.

### COG distribution

**Figure 2:**
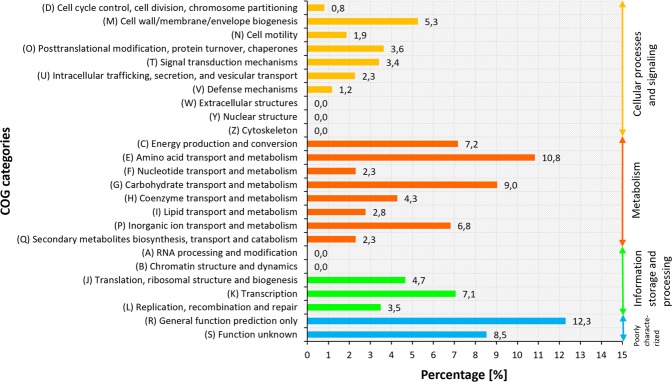
COG analysis of *Enterobacter aerogenes* LU2. Colored bars indicate the percentage of genes assigned to each COG category.

For functional studies of genome sequences, the COG database, which is a phylogenetic classification of encoded proteins, was used, and the distribution of genes within the COG categories is provided in Fig. 2 and Additional file 1^20,21^. A total of 4181 out of 4741 (88,2%) coding sequences were assigned to 20 out of 25 COGs. The largest group of coding sequences included genes involved in metabolism (1903; 45,5%), followed by poorly characterized (871; 20,8%), cellular process (771; 18,4%), and information storage and processing (636; 15,2%) genes. This analysis also showed three main functional gene classes, namely, Amino acid transport and metabolism (E), Carbohydrate transport and metabolism (G) and the General function prediction only (R) class, which are associated with basic metabolism and physiological functions, collectively constituting 32,1% of all predicted CDSs. The high percentage of genes belonging to the E and G classes as well as genes involved in the Transport and metabolism of inorganic ions (P) may suggest the innate capacity of the LU2 strain to compete with other microorganisms and survive in the cow rumen. A similar conclusion has been presented by Andrés-Barrao *et al*.^21^, who conducted their study on *Enterobacter aerogenes* SA187 in reference to the ability of this strain to survive in the rhizosphere as well as in cooperation with many different plant species. Furthermore, it was claimed that SA187 has a beneficial effect on *Arabidopsis thaliana* by providing abiotic stress tolerance. The presence of numerous genes belonging to the abovementioned classes may also suggest that the LU2 strain has a highly developed system for the transport and metabolism of a wide spectrum of C (carbon) and N (nitrogen) sources. From the perspective of studying this strain as a potential platform for the industrial production of “green chemicals” such as succinate, these results are crucial, especially considering the possibility of metabolic pathway modification of many different sugars that can be used as C sources in the culture medium. The second largest cluster consists of genes with unknown function (S) and genes involved in Energy production and conversion (E) and the Transcription process (K), representing 22,8% of all the annotated genes. The high metabolic activity of the LU2 strain is reflected in the occurrence of many genes involved in the transcription process. This state of affairs may also be related to the large number of genes encoding enzymes responsible for carbohydrate and amino acid metabolism. Interestingly, Luo *et al*.^22^ analysed 70,000 genes from 23 bacterial genomes and found that essential genes present a higher degree of evolutionary conservation than do nonessential genes. In reference to the COG classification, essential genes belonging to the G and K classes as well as to the classes of Lipid transport and metabolism (I), Translation, ribosomal structure and biogenesis (J), Replication, recombination and repair (L), and Coenzyme transport and metabolism (H) are more evolutionarily conserved, and the selection pressure has more impact on them than on the nonessential genes. In this analysis, no genes were assigned to five COG classes: Extracellular structures (W), Nuclear structure (Y), and Cytoskeleton (Z) as well as RNA processing and modification (A) and Chromatin structure and dynamics (B), belonging to the groups of Cellular processes and Metabolism, respectively. Unfortunately, as mentioned above, there is still little known about the function of 367 (8,5%) genes belonging to the S class, and 561 genes were not assigned to any COG, which is approximately 11,8% of all annotated CDSs.

### Gene ontology classification

**Figure 3:**
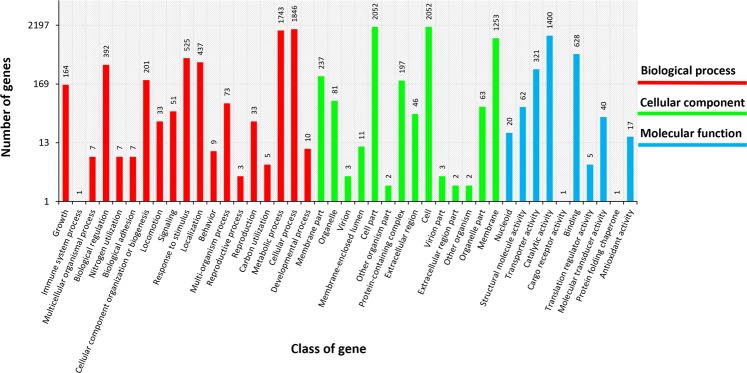
GO analysis of *Enterobacter aerogenes* LU2. Colored bars indicate the number of genes assigned to each class of genes.

To understand the putative biological functions of the classified proteins, gene ontology was investigated. A total of 14046 genes were assigned into 43 subclasses, namely, 19 subclasses of the Biological processes (BP) class, 15 subclasses of the Molecular function (MF) class and 9 subclasses of the Cellular component (CC) class at level 1 (Fig. 3, Additional file 1). Most genes have been classified into the CC class (6024; 42,89%), followed by the BP class (5547; 39,49%) and MF class (2475; 17,62%). The Cell part and Cell subclasses belonging to the CC class and Cellular process subclass belonging to the BP class have been recognized as the most abundant functional subclasses, representing 5950 genes, 42,36% of all assigned genes. These results confirmed that COG analysis indicated that genes involved in the basal metabolism of cells constitute the largest functional group. The second largest group, accounting for 31,3% of all assigned GO terms, includes the Metabolic process (1743), Membrane (1253) and Catalytic activity (1400) subclasses, belonging to the BP, CC and MF classes, respectively. For the Immune system process, Cargo receptor activity and Protein folding chaperone subclasses, only one GO term for each was assigned.

### Carbon metabolism and succinate formation pathways

The genome of *E*. *aerogenes* LU2 contains genes encoding various pathways in central carbohydrate metabolism, including the tricarboxylic acid cycle (TCA), pentose phosphate, glyoxylate, Entner-Doudoroff, and Embden-Meyerhof-Parnas pathways. Genes encoding the same biochemical pathways have also been identified in the genome of *Enterobacter* sp. SA187^21^. Such an extensive central carbon metabolism makes the LU2 strain able to metabolize various sugars and other C sources present in the rumen. Because LU2 was recognized as a highly efficient producer of SA, possible SA formation pathways have been verified. Succinate biosynthesis may be based on three metabolic pathways, namely, (i) the oxidative TCA cycle, (ii) the reductive branch of the TCA cycle, and (iii) the glyoxylate pathway, and succinate can be the end product of fermentation when sugar or glycerol serves as carbon source^23^. To check the completeness of metabolic pathways leading to succinate formation by the LU2 strain, the presence of genes involved in these pathways was evaluated. In the case of the oxidative TCA cycle, for the accumulation of succinate as a final product of fermentation, blocking the *sdh*A gene responsible for the conversion of succinate to fumarate in this cycle is necessary, so this route was not evaluated, additionally taking into account that the biosynthesis of succinate as the main product from lactose is possible under anaerobic conditions^24^. As reported by Tajima *et al*.^25^, the production of succinic acid by the strain *E*. *aerogenes* AJ110637 under anaerobic conditions was based on the reductive branch of the TCA cycle, where succinate plays the role of the H-acceptor instead of oxygen^23^. For this route, genes encoding phosphoenolpyruvate carboxylase (EC 4.1.1.31), malate dehydrogenase (EC 1.1.1.37) and fumarate dehydratase (EC 4.2.1.2) have been detected in the LU2 strain genome. Interestingly, KEGG metabolic analysis showed that there is no gene encoding fumarate reductase (EC 1.3.99.1) in the LU2 genome, a crucial enzyme responsible for the conversion of fumarate into succinate in the reductive branch of the TCA cycle. For this reason, that gene was manually found in another strain, *E*. *aerogenes* KCTC 2190, and searching against the LU2 genome using the NCBI BLAST algorithm was performed. Finally, a gene encoding fumarate reductase was identified. Another potential biochemical route for succinic acid production is the glyoxylate pathway. As reported by Cheng *et al*.^23^, under anaerobic conditions and in the absence of an additional electron donor, the glyoxylate route provides extra NADH to the fermentative pathway and consequently could enable higher yields in the succinate-producing process. Based on the KEGG map for glyoxylate and dicarboxylate metabolism, the main genes for the glyoxylate pathway that encoded citrate synthase (EC 2.3.3.1), aconitate hydratase (EC 4.2.1.3), and isocitrate lyase (EC 4.1.3.1) have also been identified in the genome, confirming that the LU2 strain has a genetic background for efficient succinate production.

### Growth and sugar transporters

**Figure 4:**
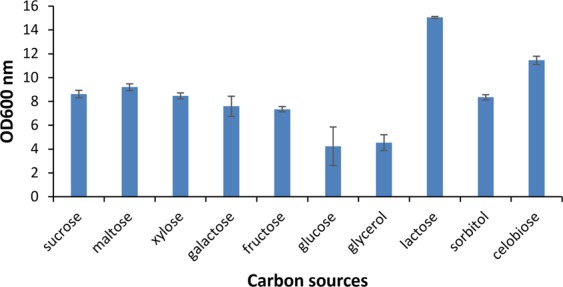
Growth of *Enterobacter aerogenes* LU2 on different carbon sources. Bars represent standard deviation. All samples were analyzed in three full biological replications.

It was recognized experimentally that the LU2 strain has the ability to grow on a wide range of industrially relevant sugars such as glucose, galactose, fructose, xylose, sucrose, maltose, lactose, and cellobiose as well as the polyhydroxy alcohols sorbitol and glycerol (Fig. 4). Phosphotransferase system components IIA, IIB, IIC, and IID, part of the phosphoenolpyruvate-dependent sugar phosphotransferase system (PTS), which is a major carbohydrate transport system in bacteria, were recognized in the LU2 strain genome for glucose, maltose, fructose, sucrose, cellobiose, mannose, mannitol, and sorbitol (glucitol)^26^. Furthermore, the PTS system was also identified for arbutin, salicin, N-acetylogalactosamine, D-glucosaminate, ascorbate and trehalose. Xylose, galactose, arabinose and ribose are taken up by the ATP-dependent ABC-type sugar transport system^27^. Lactose and gluconate are transported by the permease system and H^+^ symport mechanisms, respectively. Facilitated transporters were also recognized for 2-ketogluconate. It has already been reported that *E*. *aerogenes* has the ability to utilize glycerol^28–30^. In the LU2 strain genome, crucial genes for the glycerol uptake facilitator, glycerol-3-phosphate dehydrogenase and glycerol kinase were also detected. This analysis confirmed that the LU2 strain has the genetic capability to grow on numerous carbon sources, including glycerol and lactose, which are currently attractive and cost-effective industrial feedstocks of global interest. In addition, the identification of genes encoding carbon transporters and associated regulatory proteins indicates the possibility of metabolizing many C sources that have not yet been tested experimentally. The utilization of a wide spectrum of C sources may be related to a high number of genes encoding hydrolases, including glycoside hydrolases, which have been identified by functional analysis using the KEGG database and are presented at level 1 in Table 3. This analysis confirms the great potential of the LU2 strain as a new, efficient producer of desirable metabolites with industrial significance.

**Table 3:** Distribution of genes encoding enzymes within enzyme class (EC) based on KEGG pathways analysis.

EC number classification	EC class	Number of genes	Percentage [%]
2	Transferases	736	31,6
5	Isomerases	141	6,1
1	Oxidoreductases	502	21,6
3	Hydrolases	552	23,7
7	Translocases	79	3,4
4	Lyases	209	9,0
6	Ligases	109	4,7
**Total**		**2328**	**100**

### Anaerobic/semi-anaerobic metabolism

**Figure 5:**
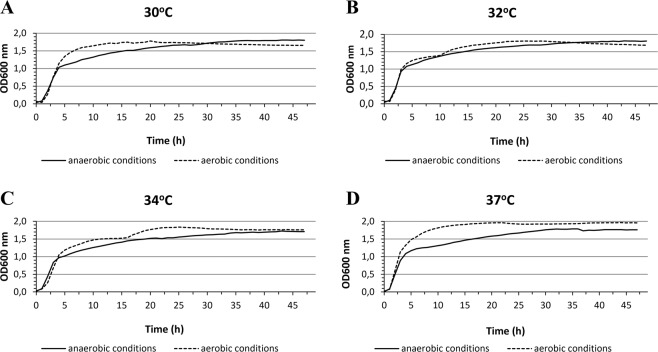
Growth of *Enterobacter aerogenes* LU2 under anaerobic and aerobic conditions at temperature of 30 ^o^C (**A**), 32 ^o^C (**B**), 34 ^o^C (**C**) and 37 ^o^C (**D**). The OD600_nm_ was measured every 1 h for 48 h. All samples were analyzed in three full biological replications.

The vast majority of industrial strains are strictly anaerobic microorganisms that require the use of rich culture media and the preservation of stringent anaerobic conditions during the multiplication of bacterial biomass and fermentation processes^31^. Meanwhile, it has been experimentally confirmed that *E*. *aerogenes* LU2 has the ability to grow under both aerobic and anaerobic conditions over a wide range of temperatures (Fig. 5). This feature makes it an attractive microorganism for industrial-scale applications because of the lack of need to maintain stringent anaerobic conditions in large bioreactors and due to the low nutrient requirements. Examination of the LU2 genome has provided information about the presence of genes involved in aerobic respiration and anti-oxidative stress, such as catalase (CAT), glutathione reductase (GR), peroxidase (POD) and superoxide dismutase (SOD), which can participate in the removal of reactive oxygen species (ROS). Due to the dual nature of the LU2 strain, the final products of fermentation may vary depending on the culture conditions. Under strictly anaerobic conditions, the LU2 strain may produce large amounts of succinate. On the other hand, under semi-aerobic conditions, the formation of 2,3-butanediol by *E*. *aerogenes* strains is also possible^32^. Regardless, we will carry out further investigations on the impact of growth conditions (aerobic/anaerobic) on the metabolite production profile.

### Antibiotic resistance genes

To predict the presence of antibiotic resistance-related genes, exploration of the LU2 genome using the ResFinder 3.1 platform was performed against “all databases”, with a selected threshold identity and selected coverage between sequences at the level of 98% and 80%, respectively. To increase the complexity of the analysis, the dataset obtained was complemented with manual BLASTp searching of resistance genes selected by Grazziotin *et al*.^33^ against the complete genome of the LU2 strain. In addition, the LU2 strain (succinic acid producer) and *E*. *aerogenes* KCTC 2190, recognized as the producer of 2,3-butanediol, were compared with each other and against the multidrug-resistant strain *E*. *aerogenes* C10 obtained from hospitalized patients (Table 2). None of the resistance genes for antibiotics belonging to aminoglycosides or macrolides or for colistin, fluoroquinolone, fosfomycin, fusidic acid, glycopeptide, lincosamide, streptogramin B, nitroimidazole, oxazolidinone, rifampicin, sulfonamide, tetracycline, or trimethoprim were identified (Additional file 1). Comparative analysis showed that only 5 resistance-related genes have been identified in the LU2 and KCTC 2190 genomes, in comparison with 15 genes that were found in the C10 strain genome. In general, 4 out of the 5 genes identified in the LU2 and KCTC 2190 strains were the same and encoded the following enzymes: ribonuclease Z/BN, Class C beta lactamase, dihydropteroate synthase type-1 and undecaprenyl-diphosphatase. Interestingly, chloramphenicol acetyltransferase 3, encoded by the *cat3* gene and conferring resistance to chloramphenicol, was found in the LU2 strain genome and was absent in the KCTC 2190 genome. Generally, the LU2 strain has a few genes associated with resistance to beta-lactam, sulfonamide (sulI), bacitracin (uppP) and chloramphenicol (cat3). However, to confirm the lack of resistance and resistance to the antibiotics mentioned above, further physiology analysis of the LU2 strain will be carried out using the BIOLOG platform.

### Bacteriocin biosynthesis

Antimicrobial compounds such as bacteriocins play an important role, especially in bacteria-bacteria interactions^34^. These secondary metabolites with a narrow killing spectrum have a crucial significance in the antagonistic activities of bacteria in their niche^35^. Based on BAGEL4, a platform that enables the mining of the bacterial genome for bacteriocins and ribosomally synthesized and post-translationally modified peptides (RiPPs), two putative clusters responsible for the production of RiPPs and other bacteriocins were identified (Additional file 1). The predicted regions, at 20,309 bp and 20,000 bp, were assigned for microcin V (colicin V) and bottromycin, respectively. The entire cluster sequence for colicin V was then compared with other sequences in the NCBI GenBank using NCBI BLAST, and the best blast hits were obtained for *Klebsiella aerogenes* AR0161 (CP028951.1) (99,63%), *K*. *aerogenes* AR0018 (CP024880.1) (99,57%) and *E*. *aerogenes* KCTC2190 (CP002824.1) (99,44%). According to Riley and Wertz^35^, colicins are the most frequently studied bacteriocins produced mainly by *E*. *coli*, which are also excellent models for evolutionary studies. The capability of colicin formation by *Enterobacter* species such as *E*. *cloacae* ENHKU01, EcWSU1, ATCC13047, and SDM, as well as by other *Enterobacter* species (*E*. *aerogenes* KCTC 2190, *E*. *lignolyticus* SCF 1, and *Enterobacter* sp. 638) was also confirmed by Liu *et al*.^34^. Moreover, all strains except *E*. *aerogenes* KCTC 2190 had genes involved in entericidin biosynthesis, and 4 out of 8 also had genes for pyocin formation. For comparison, in the LU2 strain genome, no genes for these bacteriocins have been identified, which may suggest that they are not specific to the *E*. *aerogenes* species, as they were also not detected in the KCTC 2190 strain by Liu *et al*.^34^. In the case of the cluster sequence for bottromycin, the greatest similarity was demonstrated for *K*. *aerogenes* AR0007 (CP024883.1) (99,7%), *K*. *aerogenes* CAV1320 (CP011574.1) (99,25%) and *K*. *aerogenes* AR0161 (CP028951.1), with a coverage level of 95% for all. In turn, genes responsible for the formation of this antimicrobial compound were not detected in strains tested by Liu *et al*.^34^, including the *E*. *aerogenes* KCTC 2190 strain. These results suggest that the genetic capability of the LU2 strain for microcin V and bottromycin formation is innate and not due to acquisition by horizontal gene transfer.

### Genomic islands

Mutations, rearrangements and horizontal gene transfer (HGT) are the main phenomena contributing to the continuous evolution of bacterial genomes^36^. Genomic islands (GIs) are DNA fragments acquired by host cells through HGT and integrated into chromosomes. These regions may encode numerous beneficial traits for bacteria and thus have an influence on better adaptation of microorganisms to changing environmental conditions. Due to the gene content, these regions can be divided into pathogenicity, metabolic, fitness, symbiosis and resistance islands and can be distinguished from the host genome based on different nucleotide compositions, including IS fragments, anomalous GC content, transposons and tRNA genes^37,38^. To evaluate the genetic diversity of *E*. *aerogenes* LU2, the presence of GIs acquired through HGT was identified using the IslandViewer 4 platform and the IslandPath-DIMOB, SIGI-HMM, and IslandPick prediction methods (Additional file 1)^39^. A total of 47 GIs with a length between 4,006 bp and 43,879 bp were predicted in the LU2 genome, with 15 by IslandPick (147 protein products), 23 by SIGI-HMM (183 protein products), and 9 by IslandPath-DIMOB (235 protein products). Moreover, no virulence factors and pathogen-associated genes were identified. Manual inspection of genes assigned to particular GIs showed many genes encoding transporters, including dicarboxylic acid transporters. Interestingly, the *dauA* gene, which is responsible for the aerobic transport of succinate from the periplasm to the cytoplasm at acidic pH and may play a role in regulating the metabolism of other C4-dicarboxylic acids at neutral pH, was also found in the LU2 strain genome. In addition, the *dcuA* gene, encoding an anaerobic C4-dicarboxylate transporter with succinate-fumarate antiporter activity, and the *sdcS* gene, encoding a sodium-dependent dicarboxylate transporter of fumarate, malate and succinate across the cytoplasmic membrane, have been detected. Genome analysis also showed the presence of prophage-associated genes (*IntS* and *IntA*) that play a role in the integration of the phage into the host genome. Moreover, ISs and TRs, which add to genetic diversity, were found. Many genes in the GIs acquired by HGT encode transcription factors, but many genes encoding proteins with unknown or hypothetical functions have also been detected. Surprisingly, within GIs, we have identified genes for the multidrug resistance proteins MdtH and MdtL associated with resistance to norfloxacin and enoxacin and to chloramphenicol, respectively. The *MdtH* gene was also detected in all strains mentioned above and tested by Liu *et al*.^34^, but the *MdtL* gene was absent in *E*. *cloacae* ATCC 13047 as well as in the genome of *E*. *aerogenes* KCTC 2190. These results confirm the potential resistance of the LU2 strain to chloramphenicol, as demonstrated by previous identification of the *cat3* gene.

### Genetic defence mechanism

The use of clustered regularly interspaced short palindromic repeat (CRISPR)/CRISPR-associated proteins (Cas) is one of many bacterial immunological strategies for phage attack or invasion by foreign DNA^40^. This defence mechanism plays a special role, particularly in protecting bacteria against bacteriophage invasion, which is possible during the industrial-scale production of metabolites^31^. The presence, frequency and distribution analysis of the CRISPR system in the LU2 strain genome was performed. Five CRISPR candidate lengths from 92 to 128 bp with one spacer each (24 to 40 bp in length) were determined; however, they were designated as questionable (Additional file 1). Thus, each of them was checked against the NCBI GenBank database with the NCBI BLAST algorithm. The results showed that all CRISPR candidates belong to the host species, and they were excluded as possible foreign genetic elements, e.g., from bacteriophages or plasmids. As described by Medina-Aparicio *et al*.^40^, more than 98 (43%) out of 228 complete *Enterobacteriaceae* genomes analysed lacked CRISPR/Cas systems, including 11 genomes out of all 12 tested from the genus *Enterobacter*. The results of these analyses and of our research indicate that the distribution of the CRISPR/Cas system in the *Enterobacteriaceae* family is not regular and rarely found in the genus *Enterobacter*.

### Presence of prophage sequence

One intact prophage region of length ~31,9 kb in position 26,226–58,200 with a GC content of 51,41% in the LU2 strain genome has been identified (Additional file 1). This region represents 0,63% of the entire genome size. Of all 40 ORFs predicted, 34 and 6 were classified as phage and hypothetical proteins, respectively, while no bacterial proteins in that region were found. Additionally, 1 tRNA encoding gene was annotated. The highest number of proteins in a phage most similar to those in the region was found for *Salmonella* phage RE-2010 (HM770079) using the Phaster platform, with 80% protein similarity. On the other hand, BLAST analysis using the NCBI Virus database indicated that the greatest similarity of 92% (QC, query coverage 69%) was demonstrated for *Salmonella* virus Fels2 (NC_010463), whereas *Salmonella* phage RE-2010 (NC_019488) was the second blast hit with a similarity of 92% (QC 68%). To date, there are only three reported *E*. *aerogenes* phages: F20 (JN672684), an unclassified phage UZ1 and phiEap-2 (KT287080)^41–43^. Interestingly, the prophage sequence found in the LU2 genome after BLAST alignment showed no similarity with these previously described *E*. *aerogenes* bacteriophages. However, similar prophage sequences were detected in the genomes of closely related species *E*. *cloacae* ATCC 13047 (CP001918.1), *E*. *cloacae* 704SK10 (CP022148.1) and *E*. *cloacae* ECNIH7 (CP017990.1), with similarity of 94,72% (QC 68%); 94,57% (QC 77%) and 94,63% (QC 72%), respectively. Based on the results obtained, we suppose that this is the first report on the prophage sequence of *Salmonella* virus Fels2 in the *E*. *aerogenes* genome. According to McKinlay *et al*.^1^, the presence of a prophage in the host genome has crucial significance, especially in biotechnological processes. On the one hand, the presence of a prophage allows for the use of phage-based genetic engineering. However, it can increase the possibility of phage lysis during industrial processes in bioreactors. Moreover, the presence of a prophage in the genome may increase resistance to environmental stresses, biofilm formation and supporting of horizontal gene transfer that contributes to an increase in biodiversity^44,45^. Taking into account that the LU2 strain was isolated from cow rumen, these features arising from the presence of a prophage presence may provide benefits to the bacterial host. Further studies on the effects of this prophage on the host’s physiologies should be carried out, and if the effects are negative, steps to eliminate this prophage from the genome should be taken.

### Comparative analysis of *E*. *aerogenes* LU2 with the best-known succinic acid-producing strains

**Figure 6:**
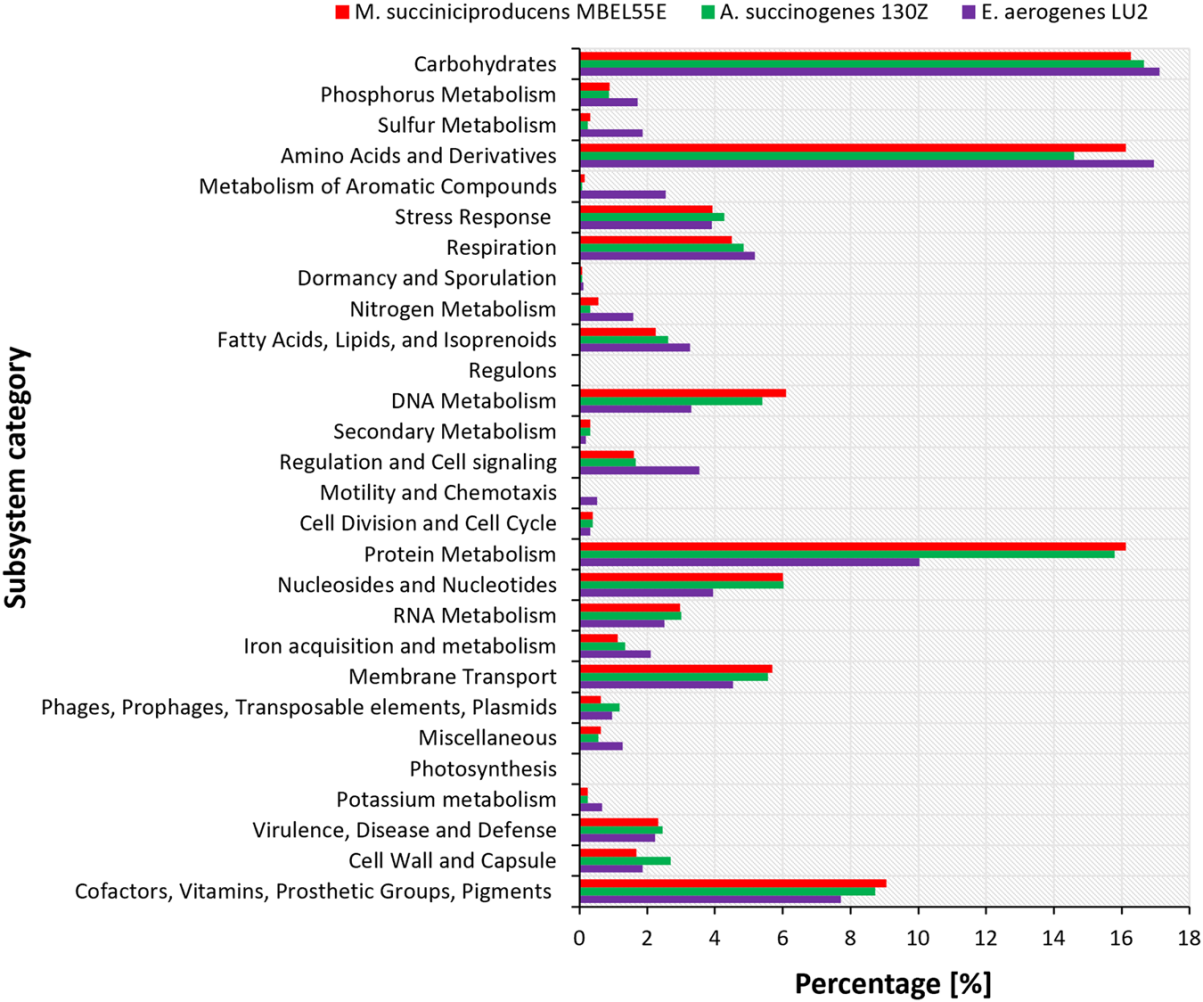
Comparison of subsystem features between *Enterobacter aerogenes* LU2, *Mannheimia succiniciproducens* MBEL55E and *Actinobacillus succinogenes* 130Z strains. Colored bars indicate the number of genes assigned to each subsystem category.

*Actinobacillus succinogenes* and *Mannheimia succiniciproducens* are known as some of the best natural succinic acid-producing bacteria^1^. Similar to the LU2 strain, they have also been isolated from the rumen and share many metabolic features. Therefore, comparative genomic analysis between *E*. *aerogenes* LU2, *A*. *succinogenes* 130Z (NC_009655) and *M*. *succiniciproducens* MBEL55E (NC_006300) was carried out. The strains *A*. *succinogenes* 130Z and *M*. *succiniciproducens* MBEL55E are closely related and have much smaller genomes than the LU2 strain, i.e., 2,319,663 bp and 2,314,078 bp with a GC content of 44,9% and 42,5%, respectively. To assess the relationships and evolutionary distance between the studied genomes, nine average nucleotide identity (ANI) values were obtained and are provided in Table 4. The ANI value for each genome that was compared with itself was expressed as 100%. Surprisingly, the ANI value for comparison of the 130Z and MBEL55E strains amounted to only 76,17%, even though both strains belong to the *Pasteurellaceae* family. Despite the fact that the LU2 genome is more than 2 times larger than the genomes of these other strains, the ANI value for the LU2 and 130Z strains was 67,44% and was not much larger than that with the MBEL55E strain. To compare the genome content of the studied strains, their genome sequences were independently uploaded to the SEED Viewer server, and gene annotation by RAST was performed (Additional file 1). Subsequently, the annotated genes were assigned and grouped into subsystem feature categories, as shown in Fig. 6. Due to the significant differences in the size of the studied genomes, the contribution of each subsystem category was presented as a percentage in relation to the entire genome. The percentage share of many subsystem categories is similar in the compared species. However, it may be noted that the LU2 strain has more genes involved in carbohydrate metabolism than the 130Z and MBEL55E strains, which constitute more than 17% of the genome. This observation confirms that the LU2 strain, similar to other *Enterobacter* strains, is characterized by the ability to utilize a wide range of C sources, a very important trait for industrial microorganisms. A clear advantage in the percentage of genes was also noted for phosphorus and sulfur metabolism as well as for amino acid and derivative subsystem categories. Nevertheless, the MBEL55E and 130Z strains possess more genes involved in membrane transport, which may represent an advantage over the LU2 strain in terms of efficient transport of important C sources that are substrates in the biosynthesis of crucial metabolites such as succinate. Moreover, in the LU2 genome, 2,5% of the genes were assigned to RNA metabolism, in comparison to 3% found in the 130Z and MBEL55E strains. *A*. *succinogenes* and *M*. *succiniciproducens* also have many more genes involved in protein metabolism (15,8% and 16,1%, respectively) than the LU2 strain does (10%), which may affect the rate of transcription, resulting in higher activity of the translational apparatus and, consequently, more efficiently protein biosynthesis. Most importantly, the LU2 strain has fewer genes assigned to the Virulence, Disease and Defense subsystem category, which confirms its great potential as a new industrial succinate producer.

**Table 4:** OrthoANI values between *Enterobacter aerogenes* LU2, *Actinobacillus succinogenes* 130Z and *Mannheimia succiniciproducens* MBEL55E.

Strains	LU2	130Z	MBEL55E
LU2	100,00	67,44	66,93
130Z	67,44	100,00	76,17
MBEL55E	66,93	76,17	100,00

## Conclusions

Hybrid sequencing based on both short and long reads allowed us to obtain a complete sequence with high accuracy in the form of one contig. Sequencing of the *E*. *aerogenes* LU2 genome provided much important information about features that may be relevant from the point of view of its potential use as an industrial strain. For example, *E*. *aerogenes* has a complete reductive branch of the TCA cycle as well as the glyoxylate pathway, which may result in higher succinic acid production efficiency^23^. Interestingly, as reported by McKinlay *et al*.^1^, *A*. *succinogenes* 130Z (ATCC 55618), a well-known succinic acid producer, lacks complete TCA and glyoxylate pathways in its genome. The presence of many genes involved in uptake and degradation pathways for a wide range of sugars is probably related to their presence in the rumen where the strain LU2 was isolated from. Moreover, these genes also grant the strain the ability to grow on many different carbon sources present in waste feedstocks, which has been experimentally confirmed. Furthermore, the strain LU2 has been shown to grow under both anaerobic and aerobic conditions in a wide temperature range, and the genetic background of this phenomenon has been investigated. COG analysis showed that the largest group of genes included those involved in the Metabolism and transport of amino acids (E), Carbohydrates (G) and genes associated with basic metabolism and physiological function (R). Similar results have been obtained for GO analysis, where the largest group of genes constituted genes involved in the basal metabolism of cells. No CRISPR/Cas systems were identified in the LU2 strain genome. It is important to note that in the genome of the LU2 strain, there is no considerable number of antibiotic resistance genes for which genes have been identified in a large number in the multidrug-resistant *E*. *aerogenes* C10 strain genome. This fact also indicates the high genetic diversity of *Enterobacter* strains. Additionally, one intact prophage region of length ~31,9 kb has been identified, which probably represents the first report on the prophage sequence of *Salmonella* virus Fels2 in the *E*. *aerogenes* genome. We believe that our research has provided numerous interesting data about a new strain from the genus *Enterobacter*, of which there is still little information in the context of the use of associated species as potential biocatalysts.

## Materials and Methods

### Bacterial strain

*Enterobacter aerogenes* LU2 was isolated from cow rumen and deposited in the International Culture Collection of Industrial Microorganisms (CCIM) in the Institute of Agricultural and Food Biotechnology under the identification code KKP 2071p. In the previous study, a new strain was recognized as an efficient succinic acid producer when lactose served as the main carbon source (manuscript in preparation).

### Strain cultivation and genomic DNA preparation

The strain was cultured under anaerobic conditions in an anaerobic jar in brain heart infusion (BHI) medium (Oxoid, UK) with the following composition (g/L): brain infusion solids (12,5); beef heart infusion solids (5,0); proteose peptone (10,0); glucose (2,0); sodium chloride (5,0); disodium phosphate (2,5); and pH 7.4 at 37 °C for 16 h. For quality assurance, genomic DNA was extracted and purified from a pure culture of a single bacterial isolate of *Enterobacter aerogenes* LU2 using the Genomic Micro AX Bacteria + Gravity kit (A&A Biotechnology, Gdynia, Poland) according to the manufacturer’s instructions (2017). DNA was quantified, and the quality was checked by measuring the absorbance at 230, 260, and 280 nm using a NanoDrop 2000 spectrophotometer (Thermo Fisher Scientific, Waltham, USA). Because long DNA fragments were required for ONT MinION, quality analysis of isolated DNA was also performed by electrophoresis in a 0,6% (wt/vol) agarose gel using SimplySafe dye (EURx, Gdańsk, Poland). DNA was visualized under UV light and archived using a GelDoc XR+ gel documentation system and ImageLab software (Bio-Rad, Hercules, USA). The size of the genomic DNA was compared to the Lambda DNA/HindIII molecular weight marker (Thermo Fisher Scientific, Waltham, USA). Only high-quality DNA samples (A260/280 = 1,8–2,0; > 5 µg; > 20 kb) were used to construct the fragment libraries.

### Genome sequencing strategy, assembly, annotation

Prior to DNA sequencing, MALDI-TOF MS analysis of the studied strain was carried out, and the mass spectra were compared with references in MALDI BioTyper 3.1 (Bruker, Massachusetts, USA) using FlexControl (Bruker, Massachusetts, USA) software. Furthermore, to confirm the protein profiles, 16S rDNA sequencing was performed, and the sequence obtained was compared against sequences deposited in the NCBI GenBank database using the NCBI BLAST algorithm^46^. Genome sequencing was performed by the Genomed SA. Briefly, a paired-end library was constructed by using the NEB-Next DNA Library Prep Master Mix Set for Illumina (NEB, Ipswich, USA) and subsequently sequenced on an Illumina MiSeq with 2 × 250 paired-end sequencing chemistry (Illumina, San Diego, USA). Furthermore, the Ligation Sequencing Kit 1D and Native Barcoding Expansion 1–12 PCR-free were used to generate a 1D long-read library (Oxford Nanopore Technologies, Oxford, UK). Purified DNA was sequenced on a MinION sequencer (Oxford Nanopore Technologies, Oxford, UK) for 24 hours. The base-calling from the nanopore sequencing data was determined using the software Albacore (Oxford Nanopore Technologies, Oxford, UK). Read trimming and filtering were performed using Cutadapt 1.9.1^47^. *De novo* sequence data assembly from both sequencing platforms was carried out using Spades v. 3.11.1 software, which resulted in one single chromosome composed of one contig with 76-fold average coverage for the Illumina MiSeq data^48^. Functional annotation of predicted genes was performed by the National Centre for Biotechnology Information (NCBI) Prokaryotic Genome Annotation Pipeline and Rapid Annotations using Subsystems Technology (RAST) Server (http://rast.nmpdr.org/)^49,50^. Genome sequences were deposited in the NCBI GenBank database in BioProject no. PRJNA516401 under accession number CP035466.

### Classification of annotated genes

Clusters of Orthologous Groups (COGs) of proteins and their functional predictions as well as gene ontology (GO) annotation were determined using the eggNOG 4.5 orthology prediction pipeline^20^. Definitions of KEGG identifiers were collected using TogoWS, and genes encoding enzymes were predicted with the Kyoto Encyclopedia of Genes and Genomes (KEGG) database^51,52^. tRNA genes were identified by tRNAscan-SE v.2.0^53^, and genes encoding rRNA were predicted with RNAmmer 1.2^54^.

### Other genome analyses

The analysis of genes involved in carbon source metabolism and transport as well as semi-aerobic and anaerobic metabolism were manually searched. The CRISPR/Cas system was identified using the CRISPRCasFinder^55^. Spacer sequences were then aligned against the *E*. *aerogenes* genome sequences deposited in NCBI GenBank using the NCBI BLAST algorithm. Phage Search Toll Enhanced Release (Phaster) was used to identify and annotate prophage sequences within the genome^56^. The presence of antibiotic resistance genes has been verified by ResFinder 3.1^57^. Genes potentially involved in bacteriocin biosynthesis were predicted with the BAGEL platform^58^. Genome features such as insertion sequences (ISs) and tandem repeats (TRs) were identified using the IS Finder Database and Tandem repeats finder program, respectively^59,60^. Genomic islands were identified using IslandViewer 4^39^. Average nucleotide identity (ANI) was calculated by OrthoANI^61^. For whole-genome sequence visualization, CGView software was used^62^.

### Growth conditions of strain LU2

The capability of strain LU2 to grow under aerobic and anaerobic conditions was determined using a Bioscreen C system (Labsystem, Helsinki, Finland). The strain was incubated in BHI (Oxoid, UK) medium for 24 h at 37 °C. Subsequently, bacterial cells were centrifuged at 12,000 rpm for 2 min and separated from the supernatant. The recovered bacterial cells were suspended in saline, and the optical density of each repetition at 600 nm (OD600_nm_) was set to 0.5 using a SmartSpec Plus Spectrophotometer (Bio-Rad, Hercules, USA). Then, 350 μL aliquots of the medium were transferred into 100-well honeycomb plates in triplicate and inoculated with 50 μL of the bacterial suspension. Anaerobic conditions were obtained by adding a few drops of sterile paraffin^63^. The experiment was conducted under aerobic and anaerobic conditions, and the OD600_nm_ was measured every 1 h for 48 h. Wells with medium alone were the control.

### Assessment of LU2 strain growth in different carbon sources

The ability to metabolize various carbon sources has been verified in batch fermentation. The strain was maintained frozen at −80 °C with 20% (w/w) glycerol added. The inoculum was cultured statically under anaerobic conditions in 100 mL bottles with gas-tight butyl rubber stoppers filled halfway by BHI (Oxoid, UK) medium for 22 h at 37 °C. Bacterial culture was then used to inoculate fermentation medium (5% (v/v)) with the following composition (g/L): carbon source (100); yeast extract (5); peptone (5); urea (2); K_2_HPO_4_ (1), MgSO_4_ × 7H_2_O (0,2); CaCl_2_ (0,5); MgCO_3_ (60). MgCO_3_ was added as a pH buffer of the fermentation broth^64^. Carbon (C) and nitrogen (N) sources were sterilized separately for 20 min at 121 °C before use and then were mixed together aseptically. The experiment was carried out in 100 mL bottles (each containing 50 mL of fermentation medium) with gas-tight butyl rubber stoppers in a rotary shaker (150 rpm) (Minitron Incubator Shaker, Infors AG, Switzerland) for 144 h at 34 °C. Removal of MgCO_3_ was performed by diluting the sample 1:1 with 7% HCl (v/v)^24^, and cell growth was verified by measuring the absorbance at 600 nm (OD600_nm_) using a SmartSpec Plus Spectrophotometer (Bio-Rad, Hercules, USA).

## Supplementary information

Supplementary Information.

## Data Availability

All data generated or analysed during this study are included in this published article (and its Supplementary Information files).
